# A New Neurosis. (Thomsen's Disease)

**Published:** 1884-09

**Authors:** William H. Spencer

**Affiliations:** Physician to the Bristol Royal Infirmary


### A NEW NEUROSIS.

### (THOMSEN'S DISEASE.)

BY

WILLIAM H. SPENCER, M.A., M.D. (Cantab),

*Physician to the Bristol Royal Infirmary.*

---

IN 1876, Dr. Thomsen, of Kappeler, in Schlesig, described a special form of disease, from which he and many members of his family suffered. The characteristic feature of the disease is described by its one essential symptom—spasmodic and tonic cramp in muscles, occurring suddenly and irregularly on attempting voluntary movement. In January, 1883, MM. Ballet and Marie published a case of this disease (*Archives de Néurologie*), naming it "muscular spasm at the commencement of voluntary movements." In March, 1883, Westphal brought two cases of the disease before the Society of Medicine of Berlin, and quoted six other cases which had come to his notice from Germany and Italy. He named it "Thomsen's Disease." Dr. Greffier published a paper on the subject in *La France Médicale* in April, 1883, and discussed it pretty fully, without, however, adding any new facts.* In July, 1883, Dr. Schönfeld, of Berlin, published a case of Thomsen's Disease, occurring in a recruit, and first recognised when the man began his drill.

About the middle of last year a man was admitted under

* This paper was reproduced in the *Glasgow Medical Journal*, November 1883, and an abstract of the reprint appeared in *The Practitioner*, February, 1884.

my care at the Bristol Royal Infirmary, who showed symptoms strikingly like those belonging to Thomsen's Disease. I am not prepared to call my case a genuine case of so-called Thomsen's Disease, but the phenomena of my case point to a neurosis of the same order. After reading what has been written about Thomsen's Disease, I have thought that the publication of my case might, perhaps, help to throw some light upon the nature of the novel and obscure set of phenomena known as "Thomsen's Disease." Some additional facts observed in my case seem to touch a question which has been raised as to the share sympathetic or vaso-motor influences take in the production of the phenomena.*

First, let me give a brief summary of what has been already set forth in regard to Thomsen's Disease.

The essential character of the disease is spasmodic rigidity or tonic contraction of muscles of the limbs, face, tongue, &c., on attempting to make voluntary movements. A patient attempting to walk upstairs could lift his legs with difficulty; after mounting seven or eight stairs, rigidity disappeared. Fingers stiffen whilst playing the piano. One leg placed in the stirrup during the act of mounting a horse, became rigid in flexion, whilst the other leg, thrown on the back of the horse, became fixed there in extension; for a short time the rider was unable to move from his ludicrous position, but presently the rigidity passed off, and he seated himself in the saddle. Children are seized in the midst of their play. Whilst eating, the masseters stiffen, fixing the jaw, as in tetanus. On attempting to rise from the sitting posture, the feet become glued, as it were, to the floor, and the knees fixed; on persevering in the effort to rise, the standing

* See *London Medical Record*, 1883, p. 190, remarks by Dr. Kesteven.

position can be assumed slowly and with difficulty; presently the muscular rigidity passes off and the patient can walk.

In some cases hypertrophy of muscles supplied by spinal nerves has been noticed, and sometimes, though not usually, the increase in bulk is associated with corresponding increase of power. The mechanical and electrical excitability of the affected muscles is said to be increased. Sensibility is not affected. Tendon phenomena are normal. The structure of the muscles remains normal.

The circumstances that provoke the attacks are various, and chiefly physical—cold, striking the foot against a pebble, strong muscular effort; and it is interesting to note that an attack may in some patients be induced by emotion, the sudden thought of the affection, or mere imagination.

The disease is said to be distinctly hereditary; it occurred in four successive generations in Dr. Thomsen's family. But it would seem that other neuroses—epilepsy, mania, &c.—alternated with the disease in question. The disease in most cases dated from infancy. In the case of Schönfeld's recruit the disease appeared at the age of fourteen, after the lad had been bitten in the leg by a dog.

As might be expected, there is great difference of opinion as to the nature of the disease. Explanation of the phenomena must be, in the nature of things, hypothetical at present. Dr. Thomsen regards the disease as a neurosis or psychosis, but this gives little information beyond expressing the opinion that we have to do with a lesion of the nervous system, and not a lesion of the muscular system. Westphal considers it a congenital anomaly of muscle-tonus; and, again, it has been proposed to call the disease "congenital muscular hypertrophy" (Jaconsiel). Ballet and Marie also adopt the muscular hypothesis. Others place the defect in the nervous system, and one observer calls the disease "spasmodic hypertrophic spinal paralysis" (Seeligmüller). Dr. Kesteven says, commenting on Westphal's explanation :—"It would seem to be more in accordance with the physiology of muscular action to look for the cause in the inhibition of the sympathetic or vaso-motor influence upon the vessels of the muscles, arresting the removal of the chemical products of muscular action, and thus interfering with nutrition and movements. In short, the muscles may be said to be approaching to the state of *post-mortem* rigidity wherein myosin becomes developed." (*London Medical Record*, 1883.) As regards treatment, everything seems to have been tried, and nothing has done any good.

Here, next, is my case.

The patient was a man, aged 36, a labourer, and married for ten years. He was under my care and observation continuously, as in-patient and out-patient, from July, 1883, to June, 1884. The history of the man up to the time of coming under my observation is as follows:— About the age of 21 he was subject to occasional attacks of sick-headache and malaise. He began to suffer from the attacks, or "fits," to be presently described, at the age of 23, and came to the Infirmary as an in-patient on account of them about a year after they first appeared. After treatment in the Infirmary he had no attacks for a year. Then, on the recurrence of the fits, he went to the Bath Mineral Water Hospital, and very shortly after this he was removed to the Gloucester Lunatic Asylum, in consequence of an attack of mania; he says he "lost his reason, and was very violent." He was in the asylum two years and nine days, and was discharged cured. I have not been able to ascertain if he had any fits during his stay at the asylum. He himself says that he has been troubled with the fits continuously since their first appearance, except during the one year, about twelve years ago, after treatment at the Infirmary. Five years ago—that is, when about 31 years of age—he met with an accident; an iron bar fell on his foot and injured his left great-toe. Eighteen months after the accident, whilst harvesting, he says he felt occasional severe pain over the occiput, and this was followed by "a lock-jaw." The trismus lasted ten days; during this time his jaws were firmly closed, and he was fed with liquids introduced between his teeth. The patient states that the condition of trismus came on gradually and slowly. Since the attack o tetanus, the "fits" have been more frequent and severe After this he seems to have attended frequently at the Infirmary as an out-patient.

As I have said, he was admitted to the Infirmary as in-patient, under my care, in July, 1883. The patient appeared to be fairly well in all respects in the intervals between his "fits." He was well nourished, had a good appetite, and slept fairly well. He had nothing to complain of beyond general weakness and loss of tone, and what occurred at the time of his attacks. His mental condition was perfectly sound. There was no history or indication of syphilis or alcoholism. A minute physical examination shewed that the *heart* was normal—the first sound, perhaps, prolonged; the *lungs* were normal; the *urine* was normal; the *patellar reflex* was normal and equal in both legs (after tapping the knee a few times, the characteristic spasm was set up in the muscles of the limb); *ankle clonus* was slightly present; the *cremasteric reflex* was just to be made out; the *plantar reflex* was not present. The reactions to the constant current, applied in the usual way, and also to Faradic electricity, were normal.

During the first period of this man's residence in the Infirmary (three months) the "fits" were accurately observed on thirty-one occasions. The attacks rarely happened singly; generally there was a succession of separate attacks for an hour, or even longer; then his motor power seemed to have become exhausted. Each separate attack lasted about two minutes, and then, after an interval of two or three minutes, was succeeded by another attack, in all respects, except intensity, similar to the preceding attacks. Taking a typical example, the order and character of the phenomena observed during a "fit" are as follows:—First in order of precedence came certain phenomena of an emotional and sensory nature: the man's appearance altered; it was clear that something was going to happen; and immediately the face became flushed, passing rapidly into a condition of intense vascular turgidity; slight tremor was now seen in the muscles of the arms and legs, followed by gradual though rapidly-succeeding muscular rigidity and spasm; the teeth were ground together; the jaws tightly clenched; the facial muscles set; the arms pressed tightly against the sides; the legs fixed in extension. When questioned, the voice was noted to be muffled and indistinct; there was no loss of consciousness at any time. Matters seeming now to have reached a climax, muscular relaxation quickly set in, and restoration to the *status quo ante*. Variations as to the details of an attack were frequently observed. Thus, amongst the initial phenomena, hiccough frequently repeated, and præcordial pain of anginal character, were observed. The attacks varied greatly in intensity; sometimes they were so severe and so frequently repeated that the patient had to keep his bed and suffered from great exhaustion; at other times, the attacks were marked by a predominance of the motor and corresponding absence of the emotional phenomena. The patient would be sitting quietly in the out-patient room; when his turn came, he would attempt to rise and walk to the table at which I sat; but the cramp would seize him in the manner characteristic of Thomsen's Disease, passing off as he persevered in his efforts to walk. Again, sitting quietly and awaiting his turn, I would address him suddenly; then the emotional phenomena, with turgidity of face, would be the first to appear, succeeded by the motor spasm.

I subjected the patient to a great variety of treatment. He took bromide and iodide of potassium, and many other things, without any relief. I gave him nitro-glycerine, with little or no effect on the anginal symptoms. The drug that seemed to produce some good effect was the Succus Conii. After taking half-ounce doses of the Succus Conii three times a day—the dose had been gradually increased to this—I find a note to the effect that the attacks had markedly decreased in number and severity. On becoming used to the drug, the good effect passed off, and I did not care to push larger doses. Subsequently, after discontinuing the Conium, I noticed, on again resuming it, that an appreciably good effect was again produced.

In the present state of nerve-pathology, and with so little experience of the affection as is now available, it would, I think, be premature to attempt to define the pathology of Thomsen's Disease. Comparison of the facts already set forth in the scanty literature of the disease with the facts of my case will, no doubt, prove suggestive enough of complexity and difficulty. It seems to me clear that Thomsen's Disease is not simply to be defined in the terms already quoted at the beginning of this paper; it is not a disease *sui generis*. We have to do with phenomena that have far-reaching and subtle alliances, both physiological and pathological, notably shewn in the alternations of other neuroses with Thomsen's neurosis in successive generations, and the occurrence of mania, pseudo-epilepsy, and tetanus in my patient, *along with* the special motor phenomena which form so striking a feature in Thomsen's Disease. The sympathetic disturbances in my case are of great interest when placed alongside of the motor disturbances. We may, no doubt, give more or less satisfactory explanations, chiefly physiological, of isolated facts and particular phenomena; but the problems presented in Thomsen's Disease have yet to be defined in the accumulation of groupings of facts or cases of a like order.

[I am greatly indebted to Dr. Fenton Evans, House-Physician at the Infirmary, for his valuable aid during the observation of this case.]

